# Efficient Extraction of Fermentation Inhibitors by Means of Green Hydrophobic Deep Eutectic Solvents

**DOI:** 10.3390/molecules27010157

**Published:** 2021-12-28

**Authors:** Patrycja Makoś-Chełstowska, Edyta Słupek, Karolina Kucharska, Aleksandra Kramarz, Jacek Gębicki

**Affiliations:** 1 Department of Process Engineering and Chemical Technology, Faculty of Chemistry, Gdansk University of Technology, G. Narutowicza St. 11/12, 80-233 Gdańsk, Poland; patrycja.makos@pg.edu.pl (P.M.-C.); edyta.slupek@pg.edu.pl (E.S.); olakramarz96@gmail.com (A.K.); jacek.gebicki@pg.edu.pl (J.G.); 2 EcoTech Center, Research Centre, Gdańsk University of Technology, G. Narutowicza St. 11/12, 80-233 Gdańsk, Poland

**Keywords:** deep eutectic solvents, extraction, furfural, 5-hydroxymethylfurfural, levulinic acid, green solvents, post-fermentation broth, hydrogen

## Abstract

The methods for hydrogen yield efficiency improvements, the gaseous stream purification in gaseous biofuels generation, and the biomass pretreatment are considered as the main trends in research devoted to gaseous biofuel production. The environmental aspect related to the liquid stream purification arises. Moreover, the management of post-fermentation broth with the application of various biorefining techniques gains importance. Chemical compounds occurring in the exhausted liquid phase after biomass pretreatment and subsequent dark and photo fermentation processes are considered as value-added by products. The most valuable are furfural (FF), 5-hydroxymethylfurfural (HMF), and levulinic acid (LA). Enriching their solutions can be carried with the application of liquid–liquid extraction with the use of a suitable solvent. In these studies, hydrophobic deep eutectic solvents (DESs) were tested as extractants. The screening of 56 DESs was carried out using the Conductor-like Screening Model for Real Solvents (COSMO-RS). DESs which exposed the highest inhibitory effect on fermentation and negligible water solubility were prepared. The LA, FF, and HMF were analyzed using FT-IR and NMR spectroscopy. In addition, the basic physicochemical properties of DES were carefully studied. In the second part of the paper, deep eutectic solvents were used for the extraction of FF, LA, and HMF from post-fermentation broth (PFB). The main extraction parameters, i.e., temperature, pH, and DES: PFB volume ratio (V_DES_:V_PFB_), were optimized by means of a Box–Behnken design model. Two approaches have been proposed for extraction process. In the first approach, DES was used as a solvent. In the second, one of the DES components was added to the sample, and DES was generated in situ. To enhance the post-fermentation broth management, optimization of the parameters promoting HMF, FF, and LA extraction was carried under real conditions. Moreover, the antimicrobial effect of the extraction of FF, HMF, and LA was investigated to define the possibility of simultaneous separation of microbial parts and denatured peptides via precipitation.

## 1. Introduction

The generation of hydrogen, optimization of the process, and purification of the gaseous products [1,2,3] are considered to be some of the key research directions for second-generation biofuels [4,5,6,7]. The most abundant fraction of lignocellulosic biomass, i.e., sugar polymers, are the source of the sole carbon source in the fermentation processes. Therefore, biomass depolymerization and saccharification are crucial for the achievement of the high efficiency of hydrogen [8,9]. However, other polymers occurring in lignocellulosic biomass, i.e., hemicellulose and lignin, are processed simultaneously during the pretreatment of biomass. Due to their chemical structure, certain compounds can be formed during pretreatment [10,11]. Consequently, the liquid phase composition is affected by this process and therefore the management of the pre- and post-fermentation broth becomes an important direction of research [5,7]. There are a number of reports [1,3,5,12] on chemical compounds formed in the liquid phase during the biomass pretreatment or after dark fermentation. Some of them may affect the efficiency of fermentation [5]. Chemical substances formed in the dark fermentation process, which are products of metabolic transformation of monosugars by microorganisms, such as organic acids, may be a source of carbon for photo fermentative organisms. Therefore, dark fermentation and photo fermentation may significantly increase the hydrogen yield if carried as subsequent processes. Among chemical compounds occurring in the liquid phase due to lignocellulosic biomass pretreatment and subsequent fermentation processes, carboxylic acids, i.e., acetic acid, lactic acid, citric acid, gluconic acid; dicarboxylic acids, i.e., succinic acid, fumaric acid, malic acid, levulinic acid; polyols, i.e., glycerin, sorbitol, xylitol and other compounds, including butanol, acetone, isopropanol, furfural, 1,3-propanediol, levulinic acid, syringole, guiacol, vanillin, and 5-hydroxymethylfurfural should be mentioned [13,14]. Nowadays, the management of the mentioned substances must be considered during the process in order to improve the efficiency of the process. Complete management of the waste stream fits perfectly into the concept of green chemical engineering and complements the technological principle of the best use of raw materials. Any work on improving the energy balance of the production of second-generation biofuels, both in terms of energy and reducing the emission of undesirable substances, leads to the development of this sector and increases the profitability of given processes. Therefore, since some of mentioned post-fermentation substances are known to be potential fermentation inhibitors, model research on inhibiting concentrations was carried out [14].

Currently, many technological processes, i.e., membrane filtration, membrane extraction, ion-exchange, solvent extraction, adsorption, or biological methods are able to separate or remove fermentation inhibitors from fermentation broth [15,16,17,18,19]. To carry out the process efficiently, it is crucial to define the nature of by-products that should be separated. Mentioned methods are considered as nonuniversal, ineffective, complicated, expensive, and time-consuming. Moreover, the possibility of the formation of by-products is mentioned [17]. The authors decided to apply solvent extraction to selectively eliminate all the problems mentioned above. However, this approach is only appropriate for selective solvents. The ideal solvent should be characterized by high selectivity to FF, HMF, and LA, nontoxicity, low vapor pressure, high stability in the aqueous phase, low viscosity, and low cost. Mostly, conventional organic solvents are used in extraction processes, including ethyl acetate, isobutyl acetate, octanol, isopropyl acetate, toluene, ethylbenzene, o-xylene, and m-xylene [20,21,22]. These solvents are characterized by the high extraction efficiency of fermentation inhibitors but are also expensive, problematic, and must include an energy-intensive recovery process. Furthermore, the most commonly used solvents are toxic and cannot be applied as green solvents. Therefore, conventional solvents should be replaced by eco-friendly ones. Therefore, the application of deep eutectic solvents (DESs) was proposed. According to the definition, DES are mixtures composed mostly of two chemical components that are able to form specific interactions between them, i.e., H-bonding or electrostatic interaction. Specific interactions between components affect the formation of eutectic liquids, which have a lower melting point compared to pure components [23,24]. DESs are characterized by favorable properties, i.e., low toxicity, cheap and simple preparation, high biodegradability, low vapor pressure, and hydrophobicity or hydrophilicity. In addition, physicochemical properties can be fine-tuned by selecting the appropriate components for DES preparation [23]. Due to these unique properties, DESs have been successfully applied for gas separation [25,26,27,28,29], water and wastewater purification, fuel desulfurization, removal of lignin from biomass, sample preparation [30,31,32], etc. However, there are little studies on the DES’ application for the extraction of fermentation inhibitors or value-added products from post-fermentation broth [33].

**Figure 1:**
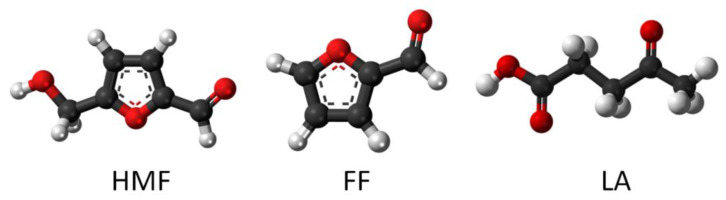
Chemical structures of fermentation inhibitors (HMF, FF, and LA). Red, white, and black colors represent the oxygen, hydrogen, and carbon atoms, respectively.

The presented research includes processing broths possibly containing chemical substances and microorganisms’ cellular parts and their remnants in the exhausted liquid phase obtained under real conditions. To purify such post-fermentation broths, a highly efficient extraction based on DES was proposed. The investigation is focused mainly on three of the mentioned compounds, i.e., 5-hydroxymethylfurfural (HMF), furfural (FF), and levulinic acid (LA), the structures of which are presented in Figure 1.

In the first part of the studies, fast screening of 56 hydrophobic DESs werer prepared using the Conductor-like Screening Model for Real Solvents (COSMO-RS). Due to the large number of possible combinations of HBA: HBD, it is impossible to test all DESs as extraction solvents. Additionally, testing a very large group of solvents is expensive and tedious. Therefore, increasing attention is being paid to the use of models that enable the preselection of solvents. Recently, one of the most popular computational models used for so-called quick screening is COSMO-RS (Conductor-like Screening Model for Real Solvents). This model can evaluate thermodynamic parameters including the solubility of individual compounds in both well-known and new solvents. Moreover, the activity coefficient can be obtained by calculating the chemical potential of molecules in a liquid solution [34,35,36,37].

DESs, which show the highest inhibitory effect on fermentation and negligible water solubility, were prepared. The main structural and physicochemical properties of the new DES were carefully studied. In the second part of the work, deep eutectic solvents were used for the extraction of FF, LA, and HMF from post-fermentation broth (PFB). Main extraction parameters, i.e., temperature, pH, and DES: PFB volume ratio (V_DES_:V_PFB_) were optimized by means of a Box–Behnken design model. To enhance the post-fermentation broth management, optimization of the parameters promoting HMF, FF, and LA extraction was carried in real conditions. Moreover, the antimicrobial effect of the conditions was applied during the extraction of FF, HMF, and LA and was investigated to define the possibility of the simultaneous separation of microbial parts and denatured peptides. To the best of our knowledge, such a detailed study on the removal of fermentation inhibitors from real samples using green DESs has not been carried or published.

## 2. Results and Discussion

### 2.1. COSMO-RS Model

**Figure 2:**
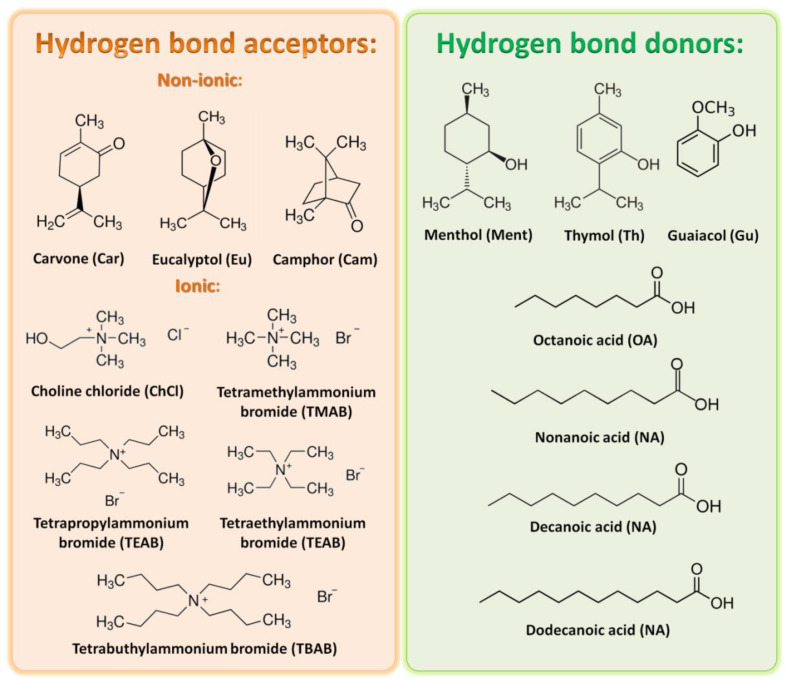
Structures of HBA and HBD used for the COSMO-RS solubility prediction.

The most important parameter that affects extraction efficiency is the solubility of extrahent in the solvent. Therefore, in the first step of the study, the fast screening of FF, HMF, and LA in the DESs was conducted using the COSMO-RS model. Due to the fact that the main fermentation inhibitors must be extracted from the aqueous phase, the water solubility in the DESs was also calculated. The screening was prepared for 56 DESs composed of ionic and nonionic hydrogen bond acceptors (HBA) (i.e., carvone (Car); eucalyptol (Eu); camphor (Cam); choline chloride (ChCl); Tetramethylammonium bromide (TMAB); Tetraethylammonium bromide (TEAB); Tetrapropylammonium bromide (TPAB); Tetrabutylammonium bromide (TBAB)), and hydrogen bond donors (HBD) (i.e., menthol (Ment); thymol (Th); octanoic acid (OA); nonanoic acid (NA); decanoic acid (DA); dodecanoic acid (DDA); guaiacol (Gu)) in a 1:1 molar ratio. The structures of HBA and HBD are presented in Figure 2.

**Figure 3:**
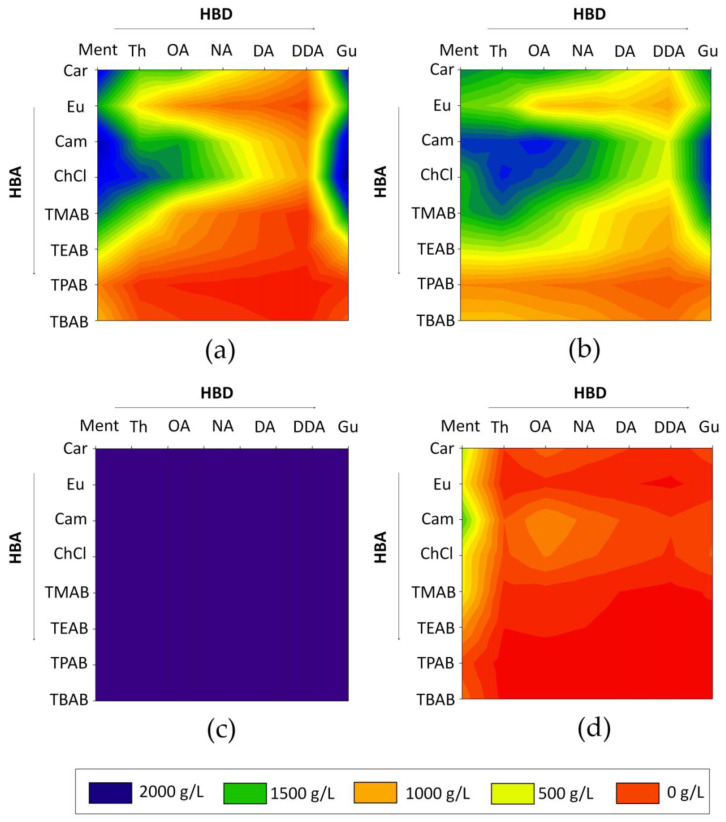
Relative solubility of the (**a**) LA; (**b**) HMF; (**c**) FF; (**d**) water in 56 deep eutectic solvent complexes predicted by COSMO-RS.

The obtained results indicate that the highest solubility of LA was obtained for Car: Ment, Cam: Ment, ChCl: Ment, ChCl: Th, ChCl: Gu, Car: Gu, Cam: Gu, ChCl: Gu in 1:1 molar ratio. For HMF, the most preferred DESs were Cam: Ment, Cam: Th, Cam: Th, Cam: OA, Cam: Gu, ChCl: Th, and ChCl: Gu. All obtained data are presented in Figure 3. Based on the calculations, it can be observed that FF is soluble in high concentrations in all analyzed DESs. On the other hand, most DESs dissolve water very poorly. It can be observed that both ionic and nonionic DESs can effectively extract fermentation inhibitors from water. However, with the increase in the number of hydrocarbon chains in HBA, the solubility of the selected fermentation inhibitors decreases. This is probably due to the fact that hydrocarbon groups do not play a major role in the formation of the strong interaction between DESs and extracted compounds. From the tested ionic HBA, ChCl showed the highest solubility of inhibitors. This is due to the fact that -OH group in ChCl structure, is able to participate in the strong hydrogen bonding formation. In all nonionic HBA, the active carbonyl (=O) group exists, which can also form H-bonds with -OH or -COOH groups in the inhibitors’ structure and HBD’s structure. A similar trend can be observed for various HBD. The highest solubility of HMF and LA was obtained for DESs composed of Gu. Guaiacol, in comparison to other tested HBD, has two active groups (=O, and -OH), which are able to perform as both hydrogen bond donors and acceptors. Therefore, Gu can efficiently interact and form H-bonding with HBA and extracted compounds. The DESs composed of the rest of HBD showed lower inhibitor solubility because of the presence of only one active group (-OH or -COOH) in their structures.

**Figure 4:**
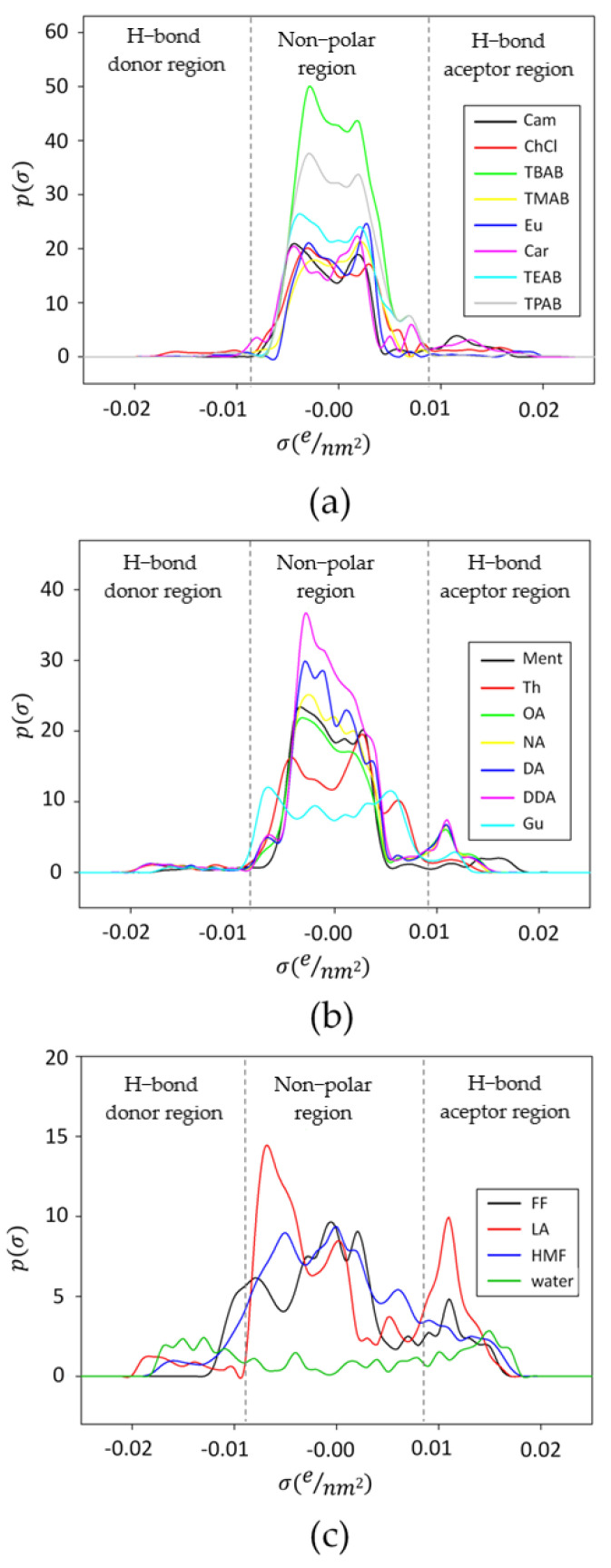
σ-profiles of chemical compounds used as (**a**) hydrogen bond acceptors; (**b**) hydrogen bond donors; (**c**) extracted compounds (FF, HMF, LA), and water. Dashed lines indicate the value ranges for the hydrogen-bond interaction (σ = ± 0.0084 e/Å^2^).

The sigma profile (σ), which is a parameter indicating the affinity of deep eutectic solvents for a molecular surface of polarity, was calculated for HBA, HBDs, and LA, FF, HMF, and water (Figure 4). The typical σ-profile diagram can be divided into three regions depending on the screening charge densities: H-bond acceptor region (σ > +0.0082 e/Å^2^), H-bond donor region (σ < −0.0082 e/Å^2^), and nonpolar region (−0.0082 < σ < +0.0082 e/Å^2^). Species of positive and negative values possess negative and positive polarities, respectively. Therefore, a molecule that can be identified in the H-bond donor region electrostatically attracts their counterparts of the hydrogen bond acceptor area due to their opposite polarities. If the peak of the molecule appeared in a charge density area below −0.0084 e/Å^2^ or above +0.0084 e/Å^2^, then this compound should be considered as adequately polar to form strong H-bonding. In addition, the narrow peak of the σ-profile indicates less polar character compared to species with a broad peak. Quaternary ammonium salts (ChCl, TMAB, TEAB, TPAB, TBAB) show high peaks in the nonpolar region. In the hydrogen bond donor and acceptor region, the σ-profiles for TMAB, TEAB, TPAB, TBAB are almost the same. Only for ChCl, the highest peak in HBD, and HBA regions can be observed. This indicates that ChCl, compared to other ionic HBA, can play a role as both a donor and acceptor of the hydrogen bond. This is most likely due to the type of anion (Cl-) and additional -OH group in the ChCl structure.

Nonionic HBA (Cam, Eu, and Car) show much more intensive peaks in the H-bond acceptor region due to the readily available acceptor group, i.e., -O- or = O in their structures. Among the group of hydrogen bond donor components, menthol and thymol show large peaks in the nonpolar region and relatively broad peaks in the H-bond region. The presence of the -OH group makes them more likely to combine with acceptor groups of fermentation inhibitors. The remaining compounds, including carboxylic acids and guaiacol, have relatively intense peaks in both the donor and acceptor regions, indicating many more hydrogen bond combinations. All the inhibitors of fermentation can form strong H-bonds with both types of DES components (HBA and HBD). Furfural has active groups -CHO, and -O-, HMF -OH, -O-, and =O, LA-COOH, and =O. Due to this, theoretically, all the proposed DES combinations should be appropriate as extraction solvents for the mentioned compounds.

Based on the theoretical COSMO-RS model, only DESs which represent the highest solubility of HMF, FF and LA and relatively low water solubility were prepared. The Car: Ment, Cam: Gu, Cam: Th, Cam: OA, C: Ment, and ChCl: Gu were selected for further research.

### 2.2. Physical Properties of DES

In the next stage, the basic physical properties of the DESs, which have a significant impact on the mass transfer process in extraction, were investigated (Table 1). The first and most important parameter is the melting point (MP). To carry out the extraction process, it is necessary to use a solvent that can be a liquid in the temperature of the process, since, from an industrial point of view, it is most preferable to carry out the extraction process at room temperature. The obtained results indicate that all of tested DES have a melting point below 0 °C. Thus, all DESs met the first condition of a suitable solvent.

The second studied parameter was the viscosity, which is highly important in many processes in which fluid flow systems are used. Most deep eutectic solvents exhibit a higher viscosity (>100 mPas) than water or other conventional organic solvents at room temperature. However, the viscosity should be as low as possible to ensure fast extraction kinetics. The obtained results indicate that all nonionic DESs have a relatively low viscosity. The highest viscosity occurred for the DES ChCl:Gu (1:3), which is in line with the previous studies [30].

In these studies, the density of DESs was also examined. The difference in density of DESs and water should be as high as possible in order to accelerate the separations of the two phases. Typically, deep eutectic solvents have densities in the range of 900 to 1350 kg/m^3^ at room temperature. The obtained results indicate that most nonionic DESs have a lower density than water (920.0–964.2 kg/m^3^), and that only Cam: Gu (1:1) has a similar density value to water. In turn, the ionic DESs have a density higher than water, 1141.37 kg/m^3^). Similar results were obtained in previous studies [25,26,33,38].

### 2.3. Analysis of Model Post-Fermentation Broth

**Figure 5:**
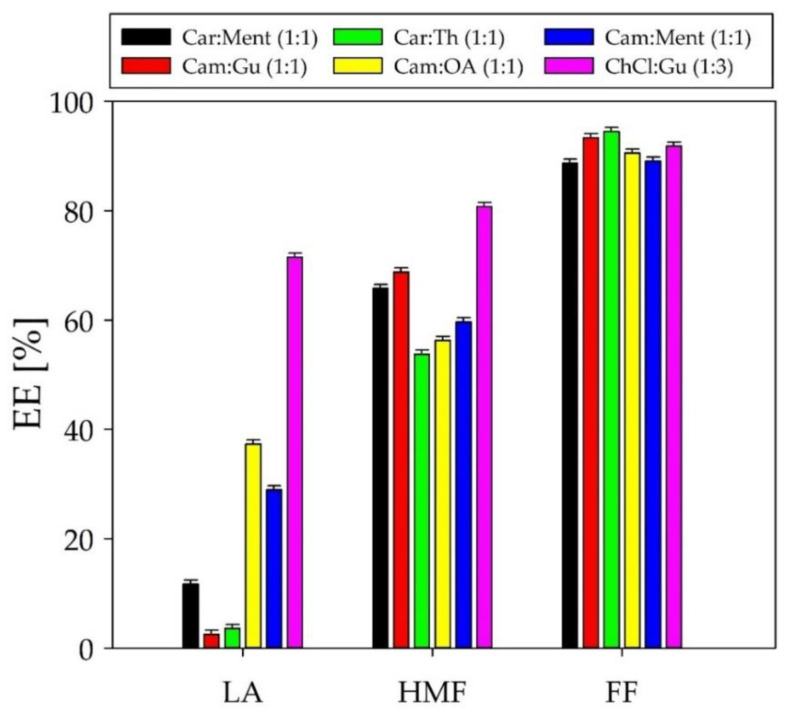
The effect of type of DES on extraction efficiency with respect to LA, HMF, and FF.

Some types of DESs have an effect on the removal of the organic compounds (such as HMF, FF, and LA) from the post-fermentation broth. Therefore, six DESs including Car: Ment, Cam: Gu, Cam: Th, Cam: OA, C: Ment in a molar ratio of 1:1, and ChCl: Gu in a molar ratio of 1:3 were used in the preliminary tests. In all experiments, the extraction conditions were constant, i.e., extraction temperature 40 °C, extraction time 50 min, pH 7, and V_DES_:V_PFB_ 1.5:1.0. The extraction tests were carried on a model PFB. Extraction efficiency (EE) was calculated using Equation (2). The results are shown in Figure 5. The obtained outcomes indicate that the highest extraction efficiency for HMF, FF, and LA was obtained for ChCl: Gu (1:3) and Cam: OA (1:1). However, ChCl: Gu (1:3) showed a relatively high solubility in aqueous PFB. This is due to the fact that pure ChCl has relatively high water solubility. Most likely, after adding ChCl: Gu to the PFB solution, DES disintegrated and part of the choline chloride dissolved in water; consequently, only guaiacol acted as the extractant. Therefore, Cam: OA (1:1) was selected as the DES for the further studies, and the results were compared with pure guaiacol. The addition of pure guaiacol to the PFB is a so-called in-situ DES generation method. In this method, pure solvent forms hydrogen bonds with individual fermentation inhibitors (FF, LA, HMF) which generates the four-component DES complex. This approach has been described in previous studies [14].

The extraction parameters such as temperature (X1), time (X2), and volumes ratio V_DES_:V_PVB_ (X3) affecting EE were optimized using the Box–Behnken design model. The Box–Behnken design matrix and responses for the percentage of extraction efficiency of FF, HMF, and LA are presented in Table 2 and Table 3 for Cam: OA (1:1) and pure guaiacol, respectively. The range of the experiment and the statistically significant variables are presented in the Materials and Methods section (please refer to Section 3.3.3).

It can be observed that, in case of all conducted experiments, the performed extraction was the most successful for FF and that the efficiency of LA was the lowest. Therefore, it should be noted that the selection of variables and their values may affect the ongoing extraction and allow to plan its main product in the given range.

The concentration of LA, HMF, and FF strongly depends on the extraction experiment profile. The dependence of the results on the certain type of processing are given in Table 4. The precise values of obtained extraction efficiency are provided in Table 3 and the optimal values of the process parameters are given in Table 4.

The determined regression equations and the results of the RSM analysis made it possible to define optimal conditions after using prognostic functions in the MiniTab 20 software. Optimization showed that the process parameters for extraction for different substances varies in the full range of the experiment. The high extraction efficiency for FF was obtained using both Car: OA: PFB and Gu: PFB. In the Authors’ opinion, the range of the experiment did not allow to define the optimal process parameters values for LA. Extraction efficiencies of 58% and 40% were obtained for Car: OA: PFB, and Gu: PFB respectively, however it is two times lower than that obtained for FF. Therefore, the applied range and solvents may be selective for FF in the experimental range. Moreover, the HMF extraction efficiency was obtained on a high level. However, for this substance, low temperature promotes high efficiency when Gu: PFB is applied and low time value promotes high efficiency when Car: OA: PFB is employed.

**Figure 6:**
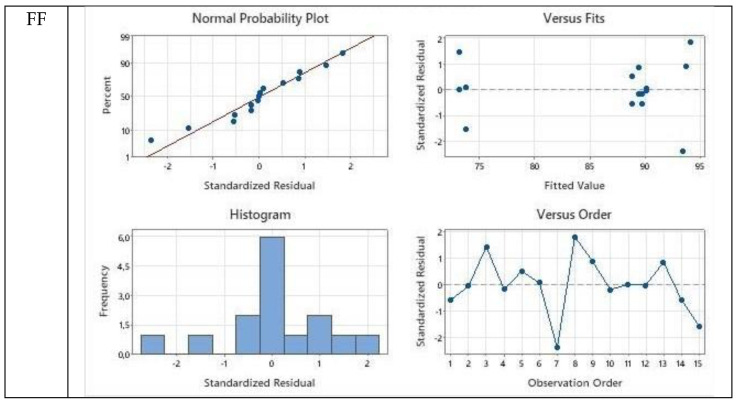

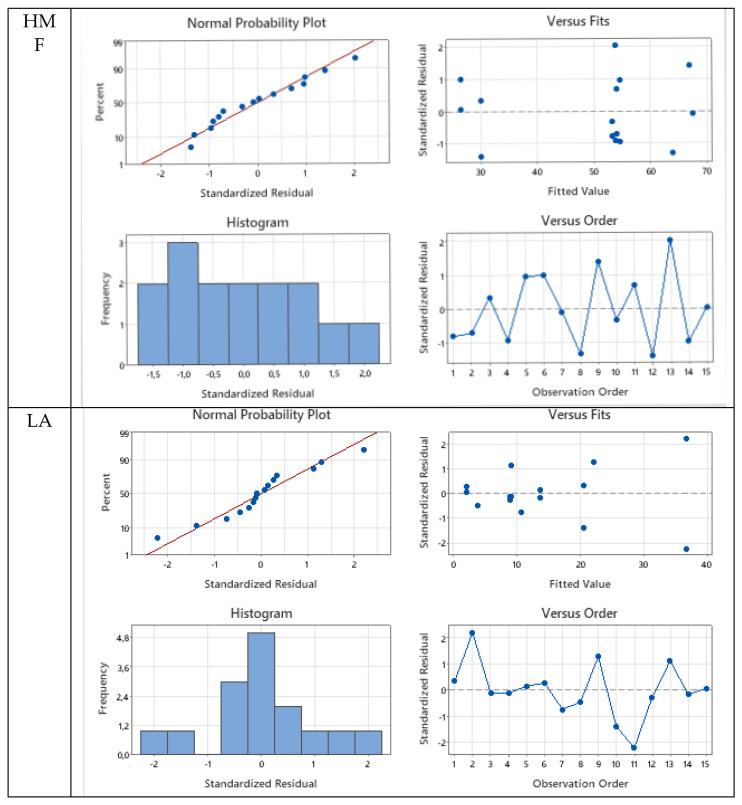
Balanced ANOVA diagrams prepared as general linear model fittings for the models obtained for FF, HMF, and LA during Cam: OA (1:1) extraction according to the RSM via the Box–Behnken design.

The interactions between model predicted and experimentally obtained values are presented as balanced ANOVA diagrams prepared as general linear model fittings. The residual plots for Cam: OA (1:1) were presented for FF, HMF, and LA with respect to normal probability plot, versus fits plot, histogram, and versus order diagrams in Figure 6.

### 2.4. Mechanism of DES Formation and Extraction

#### 2.4.1. Mechanism of DES Formation

**Figure 7:**
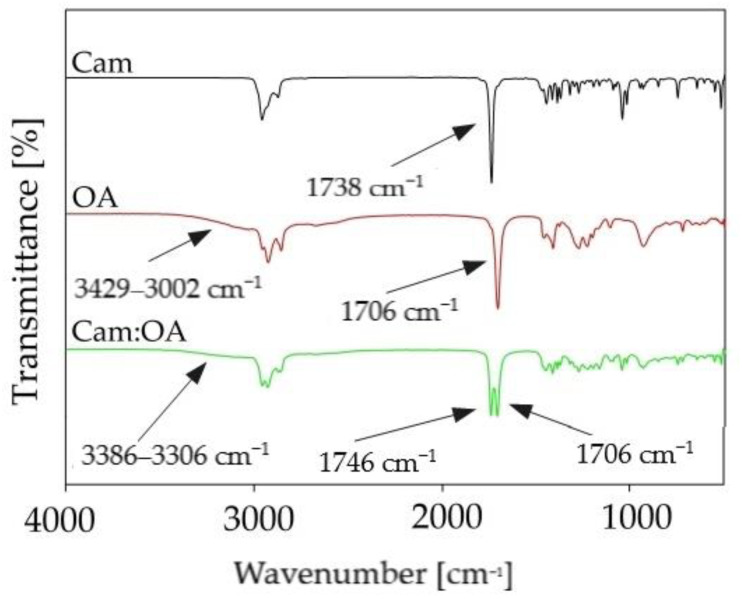
FT-IR spectra of pure components (Cam and OA) and prepared Cam: OA (1:1).

FTIR analysis was used to obtain significant information about the interactions between DES components. In the studies, analysis of the pure substances and new DESs, were performed. FT-IR spectra of pure OA, Car, and Car: OA (1:1) are presented in Figure 7. Observed shifts indicate which functional groups are involved in DES formation. In the Cam: OA (1:1) spectrum, the variable intensity, and the ranges of the wave number of the hydroxyl group from the OA can be observed. In addition, there is a shift in the group towards higher wavenumbers in the Cam: OA (1:1) spectrum compared to the pure Cam spectrum (from 1738 cm^−1^ to 1746 cm^−1^). In addition, in the FT-IR spectrum the shift of the carboxyl group in the resulting Cam: OA (1:1) can be observed (from range 3429–3002 cm^−1^ to 3386–3006 cm^−1^). It can be concluded that a hydrogen bond is formed between the -OH group from the OA as HBD and the C=O group from Cam, which is used as the HBA.

**Figure 8:**
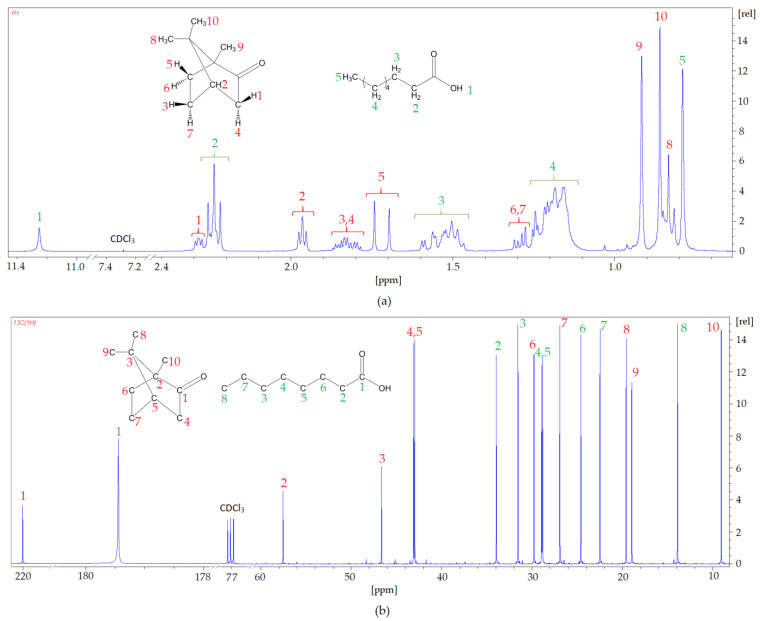
(**a**) ^1^H NMR and (**b**) ^13^C NMR spectra of Cam: OA (1:1).

In addition, to confirm the chemical structure of the newly prepared DES based on Cam: OA (1: 1) ^1^H NMR and ^13^C NMR, analyses were performed (Figure 8). In the ^1^H NMR spectrum, the shifts in signals from the protons Cam and OA can be observed. The strongest hydrogen shifts are observed for 1-H from the carboxyl group derived from organic acid OA (from 11.6 ppm to 11.25 ppm) and for both hydrogen 1-H (from 2.36 ppm to 2.29 ppm) and 2-H (from 2.09 ppm to 1.99 ppm), which are derived from the Cam phenolic ring. There are no extra peaks observed in the spectrum, which confirms the absence of by-products during DES preparation. The conducted ^13^C NMR spectra analysis produced similar conclusions. All peaks can be assigned to the respective carbon atoms from HBA and HBD, and shifts are visible, which indicates the formation of DESs. The strongest shift can be observed at the carbon atom that forms the carboxyl group in OA (from 180.81 ppm to 179.50 ppm). These shifts confirm the formation of hydrogen bonds between OA and Cam.

#### 2.4.2. Mechanism of Extraction

**Figure 9:**
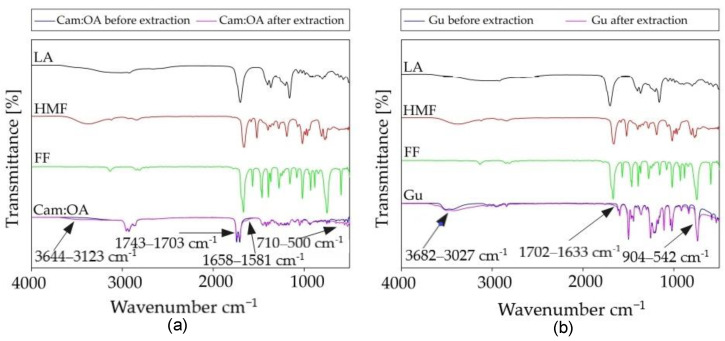
FT-IR spectra after the extraction process (**a**) for Cam: OA (1:1) and (**b**) for pure Gu.

The studies on the extraction mechanisms by Cam: OA (1:1) and Gu were performed using FT-IR and NMR spectroscopy. For this purpose, spectra of pure DES and Gu were compared with spectra of pure LA, FF, HMF, and solvents after the extraction of fermentation inhibitors from model PFB. In the FT-IR spectrum for Cam: OA (1:1) and for Gu, after the extraction process, the band corresponding to the hydroxyl group increases. The most visible shifts in Cam: OA (1:1) spectrum can be observed for the HMF. This is most likely due to the fact that HMF with the DES form stronger hydrogen bonds. The strong H-bonding can also be formed between Gu and all inhibitors. The shifts, which can be observed in the range of 3680–3027 cm^−1^ confirmed the in-situ DES formation. Similar results were also obtained in previous studies [14]. In addition, in the FT-IR spectra of Cam: OA (1:1) and Gu, shifts are visible in the ranges of around 1743–1581 and 904–500 cm^−1^, which characterize the C–H bending bonds of the aromatic band. All obtained spectra are presented in Figure 9. Visible changes confirm the effective removal of inhibitors from the PFB. 

**Figure 10:**
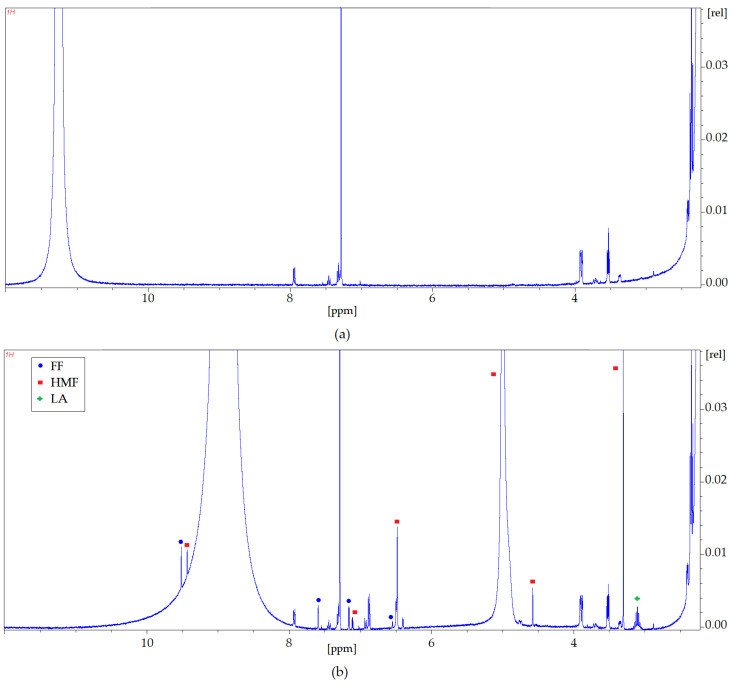
^1^H NMR spectra zoom (**a**) before the extraction process; (**b**) after the extraction process for Cam: OA (1:1).

**Figure 11:**
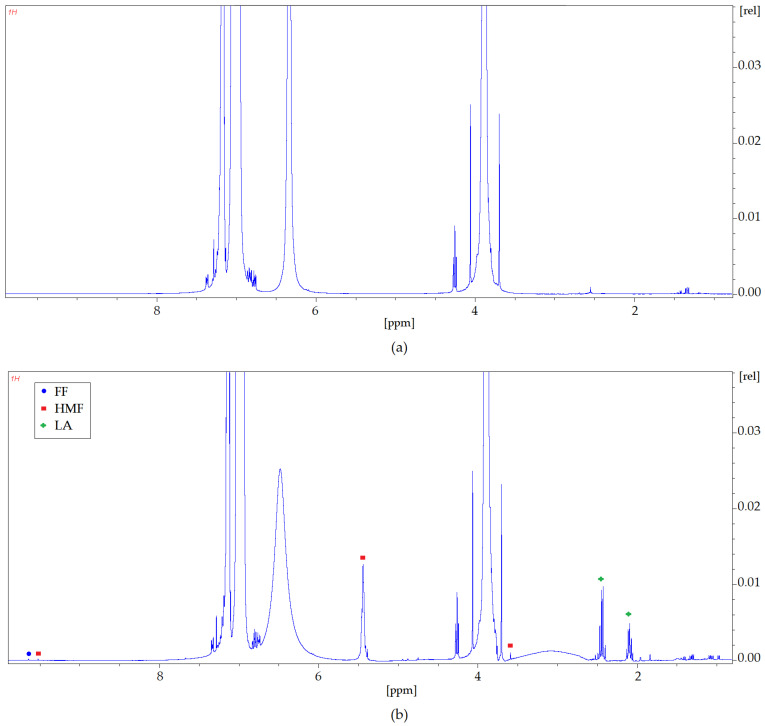
^1^H NMR spectra zoom (**a**) before the extraction process; (**b**) after the extraction process for pure Gu.

Nuclear magnetic resonance spectroscopy has been used to confirm the interaction between HMF, FF, LA, and DES (Cam: OA (1:1)) as well as Gu (Figure 10 and Figure 11). The ^1^H NMR spectra zooms were presented for pure Cam: OA (1:1) and Gu and both solvents after the extraction process. The differences observed in the NMR analyses were confirmed according to the characteristic signals of each inhibitor. Identification of the NMR signals confirms the conversion of HMF, FF, and FA into DESs. It is also noticeable that more of the signals visible in the ^1^H NMR spectrum after the extraction process come from HMF and FF. This confirms the results obtained with the FT-IR analysis. The ^1^H NMR profiles and signals also mark that the hydrogen from the hydroxyl group shifts towards lower values of ppm. This shift is most likely due to the capturing of HMF and FF by DESs, as the signals from HMF and FF are visible with the signal coming from hydrogen of the –OH group (Figure 10a).

### 2.5. Determination of Protein and Phenol Concentration Changes during Extraction

After dark and photo fermentations, loss or weakening of the integrity of the microbial cell membrane may occur due to the termination phase of bacterial growth. Moreover, changes in the growth environment may cause the release of cell contents. If the disintegration does not serve to obtain the desired material from the cells, it may be an unwanted process that limits post-fermentation broth processing.

In case of post-fermentative broth applied for extraction in the presented investigation, the effect of protein presence in the solution is not known. It is also not known whether the used DES may cause any damage in the microbial cell membrane and lead to the release of its content. Therefore, measurement was carried with respect to raw and processed material, i.e., post-fermentation broth before and after extraction, respectively. The results regarding the protein and polyphenol concentrations before and after the extraction are given in Table 5.

It can be observed that, in case of every used DES, the concentration of protein and polyphenols increased with respect to raw material samples. The proteins released during cell disintegration are generally water soluble. Some may dissolve in dilute acids or bases, others in organic solvents. Protein solubility is influenced by the concentration of inorganic salts in the solution, with a low salt concentration positively influencing the solubility of proteins. However, at higher concentrations, the solvation shell is damaged, causing the proteins to fall out of the solution. This process does not affect the protein structure, so it is reversible and is called protein salting. Proteins have the ability to bind water molecules. Due to the presence of alkali –NH_2_ and acidic –COOH groups, they are zwitterionic-dependent on the pH of the solution, and behave like acids (in an alkaline solution) or as bases (in an acidic solution). As a result, proteins can act as a pH stabilizing buffer. This property of proteins may also influence the extraction process carried out in this experiment. Therefore, it is crucial to determine the effect of certain solvents applied in the extraction of the changes occurring in the extract.

As can be seen, it was measured that, in case of every performed extraction, protein concentration increased as an effect of the process. Unfortunately, no visible trend for the protein concentration can be defined. However, due to the results, it may be stated that the DESs affect the remnant post-fermentative cells and therefore may be considered as agents, allowing to purify the post-fermentation broth from the remaining cell parts during a simultaneous process of extraction. The cell disintegration also allows to increase the polyphenols concentration in the solution and affects the extraction efficiency due to the possibility of polyphenols’ extraction from the microbial parts.

### 2.6. Purification of Real Post-Fermentation Broth

The experimental results concerning the extraction efficiency of FF, HMF, and LA from real post-fermentation broths using Cam: OA and Gu are presented in Table 6. The liquid–liquid extraction procedure was performed under optimal conditions selected by the Box–Behnken plan. For the DES based on Cam: OA (1:1), the optimal conditions were defined as follows: extraction temperature 40 °C, extraction time 30 min, and V_DES_: V_PFB_ = 1.5:1.0, whereas, for Gu, they were: extraction temperature 40 °C, extraction time 50 min, and V_Gu_: V_PFB_ = 2.5:1.0.

The obtained results indicate that both extraction solvents Cam: OA (1:1) and Gu allows one to efficiently extract LA, HMF, and LA. However, it is not possible to say unequivocally which of the solvents is preferable. The highest EE was obtained for HMF from the PFB1 sample using Gu (<99.99%). Slightly lower extraction effectivity was obtained for FF and HMF in PFB 3 and PFB 4 samples using Cam: OA (1:1). These efficiencies did not exceed 86.71%. The extraction efficiency of LA from samples in which HMF and FF were also present did not exceed 33.98%. On the other hand, in the real PFB samples, in which only LA was present, these efficiencies were equal to 70.64, and 76.45%, respectively, for Cam: OA and Gu. This is probably due to the fact that both Cam: OA and Gu create competitive H-bonding interaction with LA, HMF, and FF. However, due to the specific structures and easier access to active groups capable of creating strong interactions, HMF and FF are more readily attached to Gu and Cam: OA. In addition, the differences between solvents’ efficiency from real samples may be caused by the presence of other substances capable of forming hydrogen bonds with the solvent or by blocking active groups. Therefore, the protein and microbial remnants’ separation by centrifugation is crucial if in-situ DES formation is considered.

The comparison of the developed extraction procedure based on Gu and Cam: OA (1:1) with literature data is presented in Table 7.

The results show that the attained extraction yield for HMF, FF, and LA with the Cam: OA (1:1) application surpasses the extraction yield attained in previous papers using the DESs, ILs, and organic solvents. In addition, the research has shown that the extraction of inhibitors using pure Gu as a precursor for DES is an easy way to increase the inhibitors’ extraction yield. To the best of our knowledge, the extraction yields on the levels of <99.99% for HMF, 86.71% for FF, and 76.45% for LA are the highest yields reported to date in such a short extraction time with the application of green solvents. The literature data and presented research confirm the high dependence of the inhibitors’ extraction efficiency on the sample composition.

The proposed approach is a promising process of extraction with the application of solvents based on DES that offers several advantages when compared with conventional organic extraction solvents. However, further extraction research is needed to develop a fully established system that could compete with existing extraction processes which are performed on a larger scale. In future research, the authors want to focus on DES recovery and reuse and the performance of a techno-economic analysis in order to compare original research with the broad literature. Moreover, the hot stage microscopy analysis to confirm the deep eutectic nature of the materials shall be included in the Authors’ future research for a better understanding of the physics of heat transition and any polymorphism occurring in liquid composition in the fermentation broth. The behaviour of various deep eutectic solvents to be examined via scanning differential calorimetry would also be an interesting issue for further research for mixtures specified and selected on the basis of the ones presented in the present paper.

## 3. Materials and Methods

### 3.1. Materials

In order to begin the DES preparation and extraction process, the following reagents were used: carvone (Car); eucalyptol (Eu); camphor (Cam); choline chloride (ChCl); Tetramethylammonium bromide (TMAB); Tetraethylammonium bromide (TEAB); Tetrapropylammonium bromide (TPAB); Tetrabutylammonium bromide (TBAB); menthol (Ment); thymol (Th); octanoic acid (OA); nonanoic acid (NA); decanoic acid (DA); dodecanoic acid (DDA); guaiacol (Gu), 5-hydroxymethylfurfural (HMF), furfural (FF); levulinic acid (LA). The purity of reagents was higher than 97%. All reagents were purchased from Sigma–Aldrich (St. Louis, MO, USA). For the gas chromatographic analysis, compressed gases such as nitrogen (purity N 5.5), air (purity N 5.0) generated by a DK50 compressor with a membrane dryer (Ekom, Warszawa, Poland), and hydrogen (purity N 5.5) generated by a Precision Hydrogen 1200 Generator (PEAK Scientific, Scotland, UK) were used.

In order to determine the protein and microbial parts concentration after extraction UV-VIS spectrophotometry at 750 nm and the Folin–Ciocalteu method on Hach Lange DR 5000 apparatus was applied [44]. Determination of residual phenolic compounds was carried using the Folin–Ciocalteu method, and the results were expressed in equivalents of gallic acid. Folin–Ciocalteu reagent and gallic acid were purchased from Sigma–Aldrich (St. Louis, MO, USA), purity > 99%.

### 3.2. Apparatus

The extraction efficiency was controlled by high-performance liquid chromatography (Merck–Hitachi, Germany) with YMC-Pack ODS-2 (5 µm, 250 × 4.6 mm, YMC) column (Co, Ltd. Kyoto, Japan, Inc., Devens, MA, USA) which was maintained at room temperature. The HPLC system was connected with a spectrophotometric (UV-VIS-DAD) detector (L-7450-Merck-Hitachi, Burladingen, Germany) at wavenumber 284 nm. The chromatograms were analyzed using HSM system software (Merck–Hitachi, Germany).

The prepared DESs were structurally characterized by 1H NMR, 13C NMR Bruker Avance III HD 400 MHz (Bruker, Billerica, MA, USA) and Bruker Tensor 27 spectrometer (Bruker, Billerica, MA, USA) with an ATR adapter and OPUS software (Bruker, Billerica, MA, USA).

The proteins and microbial parts’ concentration after extraction were controlled by Hach Lange DR 5000. The spectrums were analyzed using UV Probe system software (Hach Lange, Ames, IA, USA).

Physicochemical properties of DES, i.e., density, viscosity, and melting point (MP) were analyzed using the following apparatus: a DMA 4500 M (Anton Paar, Graz, Austria); BROOKFIELD LVDV-II + viscometer (Labo-Plus, Warsaw, Poland); cryostat (HUBER, Edison, NJ, USA).

### 3.3. Procedures

#### 3.3.1. COSMO-RS Model

In the first part of studies, the conductor-like screening model for real solvents (COSMO-RS) model was used for the screening of 56 ionic and nonionic hydrophobic deep eutectic solvents for the extraction of FF, HMF, and LA from aqueous samples. DESs composed of HBA (Car; Eu; Cam; ChCl; TMAB; TEAB; TPAB; TBAB) and HBD (Ment; Th; OA; NA; DA; DDA; Gu) in 1:1 molar ratio were geometrically optimized by means of a continuum solvation COSMO model at the BVP86/TZVP level of theory. In order to find most stable DES conformers, the geometric optimization was performed in the gas phase. In the next step, the theoretical vibrational analyses were performed in order to identify the conformers which corresponded to the true energy minimum. For the conformers which were characterized by energetically favorable parameters, the full geometry optimization of DES complexes was prepared. The solubility of FF, HMF, LA, and water in DES was adopted as a parameter which has decisive influence on the efficiency of extraction. The relative solubility was calculated using Equation (1):

(1) $$log_{10}\left(x_{i}\right)=log_{10}\cdot \left[\frac{\exp\left(u_{i}^{pur}-u_{i}^{sol}-\Delta G_{i,fus}\right)}{RT}\right]$$

where:

$u_{i}^{pur}$—chemical potential of pure FF, HMF, LA, and water (J/mol);
$u_{i}^{sol}$—chemical potential of FF, HMF, LA, and water at infinite dilution (J/mol);
$\Delta G_{i,fus}$—fusion-free energy of FF, HMF, LA, and water (J/mol);
*R*—universal gas constant (8.314 J/mol⋅K);
*T*—temperature (K).

All calculations were prepared by means of ADF COSMO-RS software (SCM, Netherlands). In order to visualize the charge distribution of DES components and fermentation inhibitors, the σ-profiles were calculated using the 3D surface charge densities.

#### 3.3.2. DES Preparation and Characterization

Only DESs which represented the highest solubility of HMF, FF, and LA were prepared. The DESs including Car: Ment, Cam: Gu, Cam: Th, Cam: OA, C: Ment, ChCl: Gu were prepared by simple mixing HBA and HBD in a 1:1 molar ratio in a hotplate stirrer at 80°C until clear solutions were obtained. Then, DESs were cooled to room temperature (RT). Due to the fact that ChCl: Gu in a 1:1 molar ratio do not form an eutectic mixture, the ChCl: Gu were prepared in a 1:3 molar ratio. In the next step, for DESs which were liquid at room temperature, the structural and physicochemical properties were examined. In order to determine structural characterization, the following ATR-FTIR parameters were used: spectral range 4000–550 cm^−1^, resolution: 4 cm^−1^, number of sample scans: 256, number of background scans: 256, slit width: 0.5 cm. While, to obtain ^1^H NMR and ^13^C NMR spectra, 20 mg of DESs was dissolved in 0.7 mL of chloroform-d1 (CDCl3). Spectrophotometric measurements were made at room temperature. The main physical properties, i.e., density and dynamic viscosity were measured in the temperature range from 20 to 60 °C at atmospheric pressure. The melting point of the DESs was measured visually. In the first step, DESs were cooled to −45 °C in the cryostat. After that, the temperature was increased by 0.5 °C/min. The temperature in which DESs started to melt was taken as melting point (MP).

#### 3.3.3. Extraction Process Optimization

In the first step of research, the extraction of HMF, FF, and LA from model post-fermentation broth was performed by mixing 3 mL of DES with 2 mL of model post-fermentation broth in a 10–mL beaker. The pH of the model post-fermentation broth was 7. The extraction mixture was stirred at the temperature at 40 °C for 50 min at the magnetic stirrer at 700 RPM. Extraction conditions were used based on previous studies [30].

In the second part of the research, the extraction process optimization process was carried out for the two best DES. The authors defined statistically important variables i.e., temperature of extraction process, time of extraction process, and ratio volume DES to post-fermentation broth (V_DES_:V_PFB_). Optimization processes were carried out applying the Box–Behnken design (BBD). The BBD includes 15 experiments with different levels of three variables (Table 2 and Table 3) used to determine the influence of each of the variable parameters and their mutual interactions on the efficiency of the extraction process FF, HMF and LA.

The range of experiment was defined in Table 8. The response surface methodology (RSM) was used to determine the optimal conditions of extraction in the defined range. Box–Behnken design was applied to define the optimal parameters. The plan included 15 experiments to determine the influence of each of the variable parameters and their mutual interactions on the FF, HMF and LA extraction efficiency. The RSM methodology was conducted in MiniTab 20 software (Minitab, LLC, State College, PA, USA).

The concentration of HMF, FF, and LA in the model post-fermentation broth before and after the extraction process was determined using HPLC. The model post-fermentation broth samples before HPLC analysis were filtered through a syringe filter with a PA membrane with a pore diameter of 0.45 µm. The 20 uL of the sample was dispensed into the HPLC system. In the HPLC analysis, 1% sulfuric acid–methanol (85:15 *v*/*v*) at a flow rate of 1.0 mL/min was applied as the mobile phase. Calibration curves for HMF, FF, and LA were prepared by serial dilution of stock solution. All examined samples were within the concentration range of the standard curve (R^2^ > 0.99) and above the limit of quantification LOD: 0.0005 g/L (FF); 0.1 mg/L (LA); 0.0005 g/L; SD: (HMF); ±1.59 (FA); ±0.001 (FF); ±0.003 (LA); ±0.0002 (HMF).

The extraction efficiency (*EE*) was determined according to the Equation (2):

(2) $$EE\;\left[\%\right]=\frac{C_{in}-C_{fin}}{C{\;}_{in}}\;\cdot 100\%$$

where: 

*C_in_*—initial concentration of FF and HMF in hydrolysates [g/L];

*C_fin_*—final concentration of FF and HMF in hydrolysates [g/L].

The measurements in this study were performed in triplicates, and the statistical uncertainty in the measurement was found to be ±0.15 by weight %.

In the last step of the studies, the extraction process was conducted on real post-fermentation broth samples. Samples before the extraction process were adjusted to a pH of 7 ± 0.05 using a 1 M NaOH solution.

#### 3.3.4. Protein and Phenols Concentration Determination

The determinations were made using the developed variant of the modified Lowry method. The results were obtained using the calibration curve method. The standard substance used as model protein was ovalbumin, and the standard curve for phenolic substances was prepared against gallic acid. The total content of the sum of polyphenols was determined according to Shi et al. [45], based on the modified method of Singleton et al. [46]. The determination was carried out using the Folin–Ciocalteu (FC) reagent and a 20% sodium carbonate solution (Na_2_CO_3_). As a result of the reaction between FC, Na_2_CO_3_, and the phenolic compounds present in the tested extracts, the formation of blue complex, showing maximum absorbance at wavelength λ = 760 nm [45], occurred.

Two 10 mL volumetric flasks wrapped in aluminum foil, 4 mL of distilled water, and 0.5 mL of a standard solution of gallic acid, protein solution, or samples of extracts in the appropriate concentration were transferred and 0.5 mL of the Folin–Ciocalteu reagent was added to each flask. After one minute, 2 mL of 20% Na_2_CO_3_ was added. All flasks were made up to volume with distilled water and vigorously mixed. Thirty min after the addition of the sodium carbonate, the absorbance was measured for individual samples at a wavelength of λ = 760 nm against the blank sample, in which the tested extracts were replaced with 0.5 mL of methanol (7 mL of water + 0.5 mL of FC + 0.5 mL of methanol + 2 mL of 20% Na_2_CO_3_). The tests were repeated three times. The total sum of polyphenols was calculated from the calibration curve for gallic acid and the number of proteins from the ovalbumin calibration curve. The absorbance measurement was carried our with a Hach Lange DR 5000 spectrophotometer.

## 4. Conclusions

In the presented research, the fast screening of 56 hydrophobic DESs was prepared using the COSMO-RS model. Six DESs, including Car: Ment, Cam: Gu, Cam: Th, Cam: OA, C: Ment, ChCl: Gu, were selected due to the highest solubility of HMF, FF, and LA, and their relatively low water solubility. All tested DESs except ChCl: Gu (1:3) were stable in model and real PFB samples. In addition, the hydrophobic nature, relatively low viscosity, low melting point, and large difference in DES density compared to water confirms their potential as extracting agents. In the second part of the work, deep eutectic solvents were used for the extraction of FF, LA, and HMF from the post-fermentation broth (PFB). Main extraction parameters, i.e., type of DES, temperature, pH, and DES: PFB volume ratio (V_DES_:V_PFB_) were optimized by means of the Box–Behnken design model. DES Cam: OA (1:1) and pure Gu were selected as the most effective extraction solvents. For the DES based on Cam: OA (1:1), the optimal conditions were as follows: extraction temperature 40 °C, extraction time 30 min, and V_DES_: V_PFB_ = 1.5:1.0, whereas, for Gu: extraction temperature 40 °C, extraction time 50 min, and V_Gu_: V_PFB_ = 2.5:1.0. To enhance the post-fermentation broth management, optimization of the parameters promoting HMF, FF, and LA extraction was carried out in real conditions. Under the optimum conditions, the highest extraction efficiency for FF = 86.71%, HMF = 77.92%, and LA = 70.64% were obtained. The chemical structures of DESs and the interaction between HBA and HBD and between extraction solvents and LA, FF, and HMF were reported using FT-IR and NMR spectroscopy. Furthermore, in order to confirm the interactions, the analyses of σ-profiles were used. The obtained results indicate that hydrogen bonds play a major role in both DES formation and in the efficient removal of LA, FF, HMF from model and real PFB samples. Moreover, the antimicrobial effect of the extraction condition of FF, HMF, and LA was investigated to define the possibility of the simultaneous separation of microbial parts and denatured peptides.

The paper showed that the extraction of inhibitors from PFB is possible using DESs. The extraction procedure allows for the avoidance of high temperatures and strong acids. The introduced COSMO-RS model and the extraction procedure proved that a rational choice of the extraction solvents leads to the significant removal of inhibitors from PFB. It has also been seen to a large extent that the extraction efficiency of the inhibitors’ extraction into the investigated DESs strongly depends on the DES and PFB composition. Future research with the use of DESs and subsequent PFB is aimed at carrying out a multi-stage extraction and regeneration of the DESs used. Moreover, a subsequent removal of proteins and microbial remnants during the DES and DES precursor (i.e., Gu) is a promising direction for further research on the purification of post-fermentative broths.

## Figures and Tables

**Table 1:** Basic physical properties of DESs crucial for the efficient extraction.

DES	HBA:HBD Molar Ratio	M_W_ [g/mol]	ρ [kg/m^3^]	η [mPa s]	MP [°C]
Car: Ment	1:1	153.24	922.12	4.91	<−25.0
Cam: Gu	1:1	138.18	1026.24	7.72	<−25.0
Cam: Th	1:1	151.22	964.19	23.70	−44.0
Cam: OA	1:1	148.22	930.39	9.45	−1.4
Cam: Ment	1:1	154.25	920.20	15.30	−1.0
ChCl: Gu	1:3	128.01	1141.37	39.9 *	<−25.0

M_w_—molecular weight; ρ—density at 25 °C; η—dynamic viscosity at 20 °C, 80 RPM; MP—melting point, * dynamic viscosity at 70 °C, 70 RPM.

**Table 2:** Box–Behnken experimental design; X3 is defined as V_Cam: OA_:V_PFB_.

Run	X1	X2	X3	LA	HMF	FF
				EE %	EE %	EE %
1.	50	20	1.5	24.08	52.68	89.40
2.	30	40	1.5	57.76	53.54	90.16
3.	10	40	0.5	7.97	30.40	74.08
4.	10	20	1.5	8.02	53.14	89.40
5.	50	60	1.5	15.24	55.37	89.18
6.	30	60	0.5	4.95	27.31	73.85
7.	10	40	2.5	3.80	67.51	92.11
8.	30	60	2.5	0.04	63.04	95.06
9.	50	40	2.5	32.72	67.96	94.19
10.	50	20	1.5	6.31	53.08	89.64
11.	30	40	1.5	15.77	54.64	90.20
12.	10	40	0.5	6.23	28.92	73.15
13.	10	20	1.5	20.86	55.65	90.06
14.	50	60	1.5	12.17	53.86	88.55
15.	30	60	0.5	2.88	26.50	72.81

X1—extraction temperature, °C; X2—extraction time, min; X3—V_C:C8_:V_PFB_; for coding level, please refer to Section 3.3.3.

**Table 3:** Box–Behnken experimental design; X3 defined as V_Gu_:V_PFB_ (pure guiacol).

Run	X1	X2	X3	LA	HMF	FF
				EE %	EE %	EE %
1.	50	20	1.5	0.00	89.30	97.54
2.	30	40	1.5	0.00	90.34	98.11
3.	10	40	0.5	0.00	71.13	92.63
4.	10	20	1.5	0.00	88.03	95.97
5.	50	60	1.5	0.00	90.54	97.77
6.	30	60	0.5	0.00	74.59	93.52
7.	10	40	2.5	39.87	93.57	92.95
8.	30	60	2.5	6.56	93.18	98.18
9.	50	40	2.5	23.73	94.30	98.49
10.	50	20	1.5	0.00	86.27	95.44
11.	30	40	1.5	0.00	87.23	95.41
12.	10	40	0.5	0.00	68.26	90.42
13.	10	20	1.5	0.00	86.24	92.97
14.	50	60	1.5	0.00	92.63	95.41
15.	30	60	0.5	0.00	76.52	91.23

X1—extraction temperature, °C; X2—extraction time, min; X3—V_Gu_:V_PFB_; for coding level, please refer to Section 3.3.3.

**Table 4:** The process parameters allowing to obtain optimal values calculated via RSM analysis.

**Modeled Substance**	**T, min**	**t, °C**	**V_Car:OA_:V_PFB_**
LA	36.67	34.95	0.500
HMF	10.00	28.89	2.500
FF	41.92	20.00	2.378
	**T, min**	**t, °C**	**V_Gu_:V_PFB_**
LA	10.00	26.46	2.500
HMF	12.02	20.00	2.500
FF	45.15	60.00	2.399

*p*-value ≤ 0.05.

**Table 5:** The effect of DES on the protein and polyphenol concentration in post-fermentation broth with respect to the raw samples and samples after extraction process.

Solution	Polyphenol Concetration, mg/L, Excluding Aminoacids	Protein Concentation, mg/L
Raw LA	0.026 ± 0.002	0.051 ± 0.001
Raw FA	0.107 ± 0.001	0.044 ± 0.001
Raw FF	0.022 ± 0.002	0.044 ± 0.002
Raw HMF	0.018 ± 0.002	0.054 ± 0.004
Cam:OA + model PFB	0.045 ± 0.003	0.251 ± 0.023
Cam:OA + PFB1	0.043 ± 0.001	0.194 ± 0.017
Cam:OA + PFB2	0.039 ± 0.007	0.219 ± 0.022
Cam:OA + PFB3	0.051 ± 0.002	0.625± 0.034
Cam:OA + PFB4	0.046 ± 0.001	0.251 ± 0.025
Cam:Gu + model PFB	0.037 ± 0.001	0.198 ± 0.016
Gu + model PFB	0.042 ± 0.002	0.233 ± 0.028
Gu + PFB1	0.039 ± 0.004	0.165 ± 0.018
Gu + PFB2	0.306 ± 0.002	0.173 ± 0.017
Gu + PFB3	0.061 ± 0.001	0.845 ± 0.048
Gu + PFB4	0.052 ± 0.002	0.098 ± 0.013
Cam:Ment + model PFB	0.043 ± 0.002	0.229 ± 0.032
Car:Ment + model PFB	0.039 ± 0.001	0.512 ± 0.027
ChCl:Gu + model PFB	0.045 ± 0.004	0.744 ± 0.042
Cam:Th	0.051 ± 0.002	0.358 ± 0.004

**Table 6:** The extraction efficiency of FF, HMF, and LA from real post-fermentation broths (PFB) using Cam: OA (1:1) and Gu.

PFB	Inhibitors	Cam: OA [EE %]	Gu [EE %]
1	LA	12.68	13.62
	HMF	77.92	<99.99
	FF	78.88	21.05
2	LA	70.64	76.45
3	LA	9.95	10.02
	FF	86.71	81.66
4	LA	11.41	33.98
	FF	43.42	37.10
	HMF	64.66	60.62

**Table 7:** Comparison of the developed extraction procedure based on DES with other solvents.

Extraction Solvent	V_Solvent_:V_HY_	Extraction Time [h]	Temp. [K]	Extraction Yield of HMF [%]	Ref.
**HMF**					
Isobutyl acetate	1:3	4	298	76.6	[21]
Toluene	1:1	2	298	16.7	[20]
1-Hexyl-3-methylimidazolium hexafluorophosphate ([C6mim][PF6])	1:5	0.5	298	83.0	[39]
-n-tetraoctylammonium bromide: decanoic acid (1:2) (TOABr:DecAc (1:2))	1:1	2	298	69.7	[20]
	n.d.	2	303	75.0	[40]
Thymol: decanoic acid (Th:DecAc (1:1))	n.d.	2	303	80.0	[40]
Thymol:Lidocaine (2:1) (Th:Lid (2:1))	n.d.	2	303	85.0	[40]
Camphor: 1-decanol (C:DOL (1:2))	1.5:1	0.66	313	87.9	[33]
**FF**					
Isobutyl acetate	1:3	4	298	98.1	[21]
Toluene	1:1	2	298	83.0	[20]
1-Hexyl-3-methylimidazolium hexafluorophosphate ([C6mim][PF6])	1:5	0.5	298	76.0	[39]
-n-tetraoctylammonium bromide: decanoic acid (1:2) (TOABr:DecAc (1:2))	1:1	2	298	85.0	[20]
Tetrahexylammonium bromide:decanoic acid (1:3) (THABr:DecAc (1:3))	1:1	4	323	85.0	[41]
Tetrahexylammonium bromide: dodecanoic acid (1:3) (THABr:DoDecAc (1:3))	1.5:1	4	323	85.0	[41]
Tetraoctylammonium bromide:decanoic acid (1:3) (TOABr:DecAc (1:3))	1.5:1	4	323	80.0	[41]
Tetraoctylammonium bromide:dodecanoic acid (1:3) (TOABr: DoDecAc (1:3))	1.5:1	4	323	80.0	[41]
Camphor: 1-decanol (C:DOL (1:2))	1.5:1	0.66	313	79.2	[33]
**LA**					
Dodecane	1:1	2	298	9.34	[42]
Benzene	1:1	2	298	6.02	[42]
1-octanol	1:1	2	298	28.57	[42]
4-methyl pentan-2-one (MIBK)	1:1	2	298	41.07	[42]
Dichloromethane (DCM)	1:1	2	298	15.18	[42]
trioctylphosphine oxide:Decanoic Acid (1:1) (TOPO:DecAc (1:1))	1:1	1	298	82.32	[43]
	1:1	1	313	65.29	[43]
trioctylphosphine oxide: Dodecanoic Acid (1:1) (TOPO-DoDecAc (1:1))	1:1	1	298	65.87	[43]
	1:1	1	313	64.52	[43]
trioctylphosphine oxide (TOPO)	1:1	1	313	80.37	[43]

**Table 8:** The range of experiment and the statistically significant variables.

Variable	Unit	Symbol	Coding Level		
			−1	0	1
temperature	°C	X1	20	30	60
time	min	X2	10	30	50
V_DES:_V_PFD_	%	X3	0.5	1.5	2.5

## Data Availability

Not applicable.

## Sample Availability

Samples of the compounds are not available from the authors.
